# Effectiveness of rescue Me CPR! smartphone app providing real-time guidance to untrained bystanders performing CPR

**DOI:** 10.1016/j.heliyon.2023.e20908

**Published:** 2023-10-12

**Authors:** Brennan P. Marsh-Armstrong, Eri Seng, Fan Ting-Wei, Stella Saka, Mark Greenberg

**Affiliations:** University of California San Diego, La Jolla, CA, 92037, USA

**Keywords:** Cardiopulmonary resuscitation, Cardiopulmonary resuscitation guidance application, Medical simulation, Smartphone application, Chest compression fraction, Rescue breath, Naloxone, Opioid overdose, Defibrillation

## Abstract

**Background:**

Out-of-hospital cardiac arrest (OHCA) is a persistent global health challenge, owing, in part, to low rates of population CPR training. Smartphone applications have the potential to widely disseminate CPR basic training to a populace, but other studies have found multiple limitations in previously developed CPR guidance applications (CPR-GA). This study aims to use medical simulation to assess the relative CPR performance of novices using the ‘Rescue Me CPR!’ (RMC) app, a custom CPR-GA designed by this research team, to novices using ‘PG-CPR!’ (PGC), the most downloaded CPR-GA available in the USA, and to CPR certified medical personnel.

**Methods:**

In a prospective randomized experimental trial of 60 individuals, subjects were either given the RMC app, the PGC app, or had active CPR certification. They were presented a cardio-pulmonary arrest scenario and were observed while performing CPR on a high-fidelity manikin. Data was collected through four cycles of CPR, during which time 24 pertinent performance metrics and CPR steps were timed and recorded. These metrics were assessed on their own and used to calculate average time to compressions, average chest compression fraction, and rate of high-quality CPR for each study group.

**Results:**

CPR certified subjects called 911 in 100 % of simulation cases, started compressions 34 ± 10 s after first seeing the simulated patient, had an average chest compression fraction of 0.52, and performed high-quality CPR in 25 % of aggregate compression cycles. PGC app users called 911 in 70 % of simulation cases, started compressions 86 ± 17 s after first seeing the simulated patient, had an average chest compression fraction that could not be assessed due to inconsistent pauses during CPR, and performed high-quality CPR in 2.5 % of aggregate compression cycles. RMC app users called 911 in 100 % of simulation cases, started compressions 55 ± 6 s after first seeing the simulated patient, had an average chest compression fraction of 0.48, and performed high-quality CPR in 50 % of aggregate compression cycles.

**Conclusion:**

The results of this study demonstrate that in all studied metrics, except time-to-first-compression, CPR provided by individuals using the RMC app is statistically equivalent or superior to CPR performed by a CPR certified individual and, in almost every metric, superior to CPR performed by users of the most downloaded android CPR guidance application, PG-CPR.

## Highlights

• Rescue Me CPR a phone app was designed to guide the untrained during an arrest.
• When compared to trained providers, is equally effective providing resuscitation.
• Rescue Me CPR users are more likely to request a defibrillator or naloxone.
• The app coaches the novice with voice, text and video in real-time.
• This technology may be the solution to deploying CPR on a mass scale.

## Abbreviations

CPR: cardiopulmonary resuscitation
OHCA: Out-of-hospital cardiac arrest
CPR-GA: CPR guidance applications
RMC: ‘Rescue Me CPR!’
PGC: ‘PG-CPR!’
AHA: American Heart Association
AED: (automated external defibrillator)

## 1 Introduction

### 1.1 Background and importance

Modern cardiopulmonary resuscitation (CPR) began in 1960 as a cumulative result of William B. Kouwenhoven's research, building upon centuries of prior data [1]. In 1963 the American Heart Association (AHA) endorsed the technique, and in 1966 standardized CPR guidelines were established [2,3]. Since then, the technique has received iterative evidence-based improvements. These include but are not limited to the first unified international guidelines in 2000, modifications to the pace and number of chest compressions, variant instructions for pediatric and airway obstruction scenarios, and use of AED's (automated external defibrillators) when available [[2], [3], [4]]. A full description of up-to-date guidelines is published by the AHA directly [5].

Out-of-hospital cardiac arrest (OHCA) remains a global public health challenge, with nearly 4 million cases annually and an expected rate of survival without neurologic deficits under 10 % [[6], [7], [8]]. Factors linked to higher rates of survival (as much as 50 % in some studies) and reduced neurologic complications include early initiation of CPR, high-quality CPR performance, early defibrillation, and early administration of epinephrine [[9], [10], [11], [12], [13], [14]]. On average, CPR performed by trained bystanders increases survival rate of OHCAs 2–4 fold [15,16]. Despite high prevalence of OHCA in the USA and the demonstrated benefits of well-performed CPR, annual population training rates are only 2.4 % [17]. This occurs despite countless programs, including mandatory schoolchildren training, lay public education programs, and media campaigns [18,19].

Owing to their ubiquity, smartphone applications are an ideal way to disseminate education and guidance on CPR. Within the USA Android and iOS app stores, there are many proactive teaching CPR apps but few designed to interactively assist real-time in CPR. We defined the latter here as CPR-guidance applications (CPR-GA). While there have been studies done on the effectiveness of international CPR training smartphones applications, none, to our knowledge, have been conducted on applications available in the USA [[19], [20], [21], [22], [23]]. Studies have repeatedly shown that CPR-GAs facilitate effective or near-effective CPR, but at a critically delayed pace [[19], [20], [21], [22], [23]]. We have previously detailed the utilization of medical simulation to develop ‘Rescue Me CPR!‘, an AHA-compliant CPR-GA that uses simultaneous text, audio, and visual teaching specifically designed to minimize delay before first CPR compression [24].

### 1.2 Goals of this investigation

To assess and report on the effectiveness of ‘Rescue Me CPR!’ as a potential guidance tool for facilitating CPR performed by untrained people, we used medical simulations to study relative performance metrics of CPR performed by novices using the ‘Rescue Me CPR!’ app, novices using the most downloaded CPR-GA available within the USA (PG-CPR), and CPR certified medical workers.

## 2 Methods

### 2.1 Study setting and design

This study was conducted at the University of California San Diego Simulation Center and was approved by the university's Institutional Review Board (Approval Number 804734). Data was collected between September 2022 and May 2023. All subjects were over 18 years old and provided informed written consent prior to testing. Other than name, no identifying, contact, or demographic information on subjects was recorded. Subjects consisted of a control group of 20 CPR-certified (within the last 2 years) medical students and 40 untrained subjects randomly assigned to either the ‘PG CPR’ (PGC) or ‘Rescue Me CPR!’ (RMC) groups, who used those respective apps during the experiment.

**Fig. 1:**
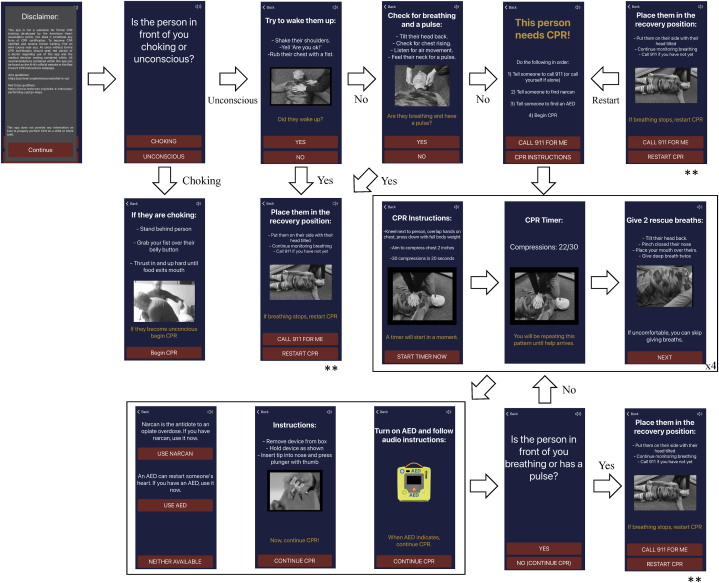
Workflow of the ‘Rescue Me CPR!’ smartphone application. Each app view has simultaneous redundant text, video, and audio instructions. The portions of the app roughly fall into patient assessment, compression/respirations, reassessment, with the latter two cycling until the patient becomes responsive. All three views marked by ** are the same location within app flow.

The top 50 smartphone applications displayed on the android's Google play store when searching for the terms “CPR,” “cardiopulmonary resuscitation,” “BLS,” and “basic life support” were sampled for CPR-GAs, defined as applications pertaining to the topic of CPR that are meant to be used during active CPR, intended for use by novices rather than medical professionals, and not meant to accompany another commercial product. The PGC application was the most downloaded application that met this criteria and was thus chosen for this study [25]. The RMC is a CPR-GA custom designed by the research team to both strictly follow AHA guidelines and minimize time to first compression [24,26]. It provides simultaneous step-by-step written, audio, and visual guides on the process of performing CPR and was optimized to minimize user decision-making. The app design and its workflow are visualized in Fig. 1. Subjects in the abovementioned experimental groups were provided with a Samsung Galaxy S8 smartphone with the appropriate application preloaded on it. The control group was not provided with a smartphone for use during the study.

After the consenting process and obtaining the appropriate smartphone application, subjects were taken to a prepared simulation room at the UCSD School of Medicine Simulation Center. Placed on the floor inside the room there was a Laerdal SimMan 3G high fidelity manikin [27]. This manikin can breathe, blink, have a pulse, and reports whether chest compressions are performed at 100-120bpm and with greater than 2 inches of compression. When the study began, COVID-19 precautions were in place, so a replicable airtight plastic barrier was placed over the manikin's mouth and nose. Immediately prior to testing each subject, the manikin's face and torso were cleaned with CaviWipes antiseptic wipes and the mouth cover was replaced. The manikin was remotely connected to a laptop in the corner of the room from which the researcher controlled the manikin and recorded data. Before entry, subjects were provided with a written and verbal prompt describing the subsequent scenario and given the chance to ask any questions. This prompt is included as Supplemental 1 and roughly instructs subjects to use the resources and knowledge they have to save a person they find unconscious on the floor who may or may not be breathing.

**Fig. 2:**
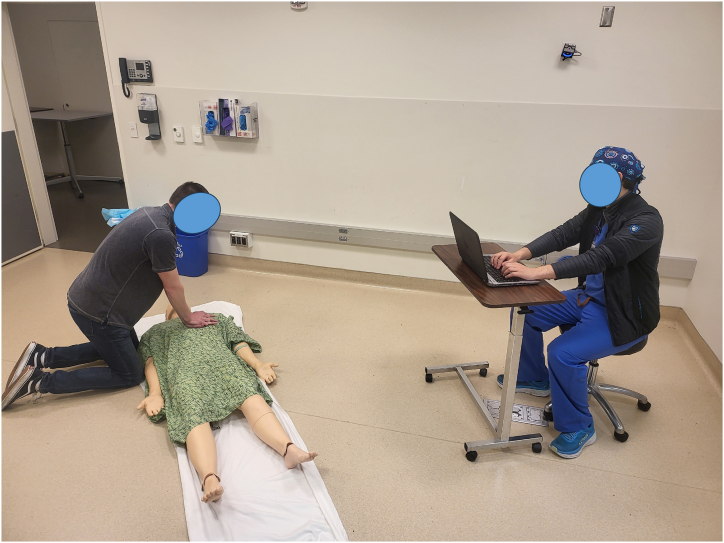
Experimental setup. Subjects were read a prompt describing the situation outside the door. Inside the room was a SimMan 3G placed on the ground and connected to the data collection computer. At the start of the simulation, the manikin is not breathing, has no pulse, and is unresponsive. When subjects enter the room, a timer and data collection are started. The sequence then has the patient assessed and completes four cycles of compressions before the manikin's vital signs return. The experimenter observed from the desk and provided the subject verbal confirmation that 911 had been called and Narcan was provided, when appropriately requested by the subject.

A timer began when subjects entered the simulation room (Fig. 2). The manikin initially had no pulse and was not breathing. Subjects proceeded to assess the simulated patient's status and provide appropriate basic life support, using the respective app provided to guide them.

After the subjects identified that the manikin required CPR, the simulation proceeded through 4 cycles of compressions, with or without rescue breaths (per AHA guidelines). If the subject had asked to call 911 or attempted to do so with the app itself, they were notified emergency services were on the way two cycles of CPR later. The version of the app they were provided had the 911 auto-dial feature disabled. If the subject had requested nasal naloxone in the simulation, they were given a mock nasal applicator of the medication during the fourth cycle of compressions. After the 4th cycle of compressions, the manikin's pulse and respirations returned, so the subjects were able to identify the change in status. The simulation ended when they stopped actively assisting the mock patient and/or when verbally prompted, stating there was no further care needed. If the subject started another cycle of CPR, the manikin began audibly groaning and the study proctor stated that an ambulance had arrived, ending the simulation.

### 2.2 Data collection and processing

**Fig. 3:**
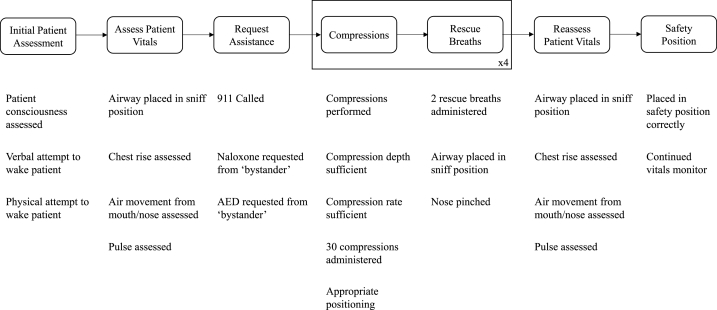
Variables collected during experimentation, categorized by the phase of the simulation when they were collected. In all cases, whether and/or when the subject performed a given task was recorded. Automated external defibrillators (AED) were requested but not used in this simulation. Sufficient depth was assigned if at least 28 compressions in a set were >2 cm (reported by SimMan 3G). Sufficient rate was assigned if compression rate was reported by the SimMan 3G as between 100 and 120 for at least 28 compressions in a set. Correct compression number was assigned if 29–31 compressions were given in a set. Appropriate positioning was given if subject was on their knees, their hands had overtopped one another, and their shoulders were roughly aligned over their wrists.

While subjects were performing this task, the study proctor recorded the timing and values of metrics shown in Fig. 3. CPR compression rate and depth were assessed by the SimMan 3G's integrated sensors and accompanying software. Delivery of respirations was assessed via the subject's cheek puffing, indicating both a lip seal and delivery of gas volume restricted by the protective barrier. Other metrics were visually assessed. Timing of the many simultaneous metrics was accomplished with a custom data collection interface designed for this study and written in Python 3.10 primarily using the Tkinter and Numpy packages.

### 2.3 Statistical analyses

RStudio was used for all statistical analysis, primarily ANOVA and *t*-test assessments of the relationship of CPR performance metrics between test groups, for all variables in Fig. 3. Chest compression fraction (CCF) is the proportion of time spent performing compressions compared to the total duration of the resuscitation [28]. High-quality CPR in compliance with AHA guidelines can be considered that which includes 30 compressions administered at a rate between 100 and 120 compressions per minute, compressing the chest >2 inches, a chest compression fraction of 0.8 or above, proper hand positioning, and two rescue breaths administered that cause chest rise (or this step intentionally omitted) [29]. Both CCF and rate of high-quality CPR were calculated for each subject and analyzed in a similar manner. All reported p-values compare either of the experimental app-using groups to the control CPR-certified group.

## 3 Results

**Fig. 4:**
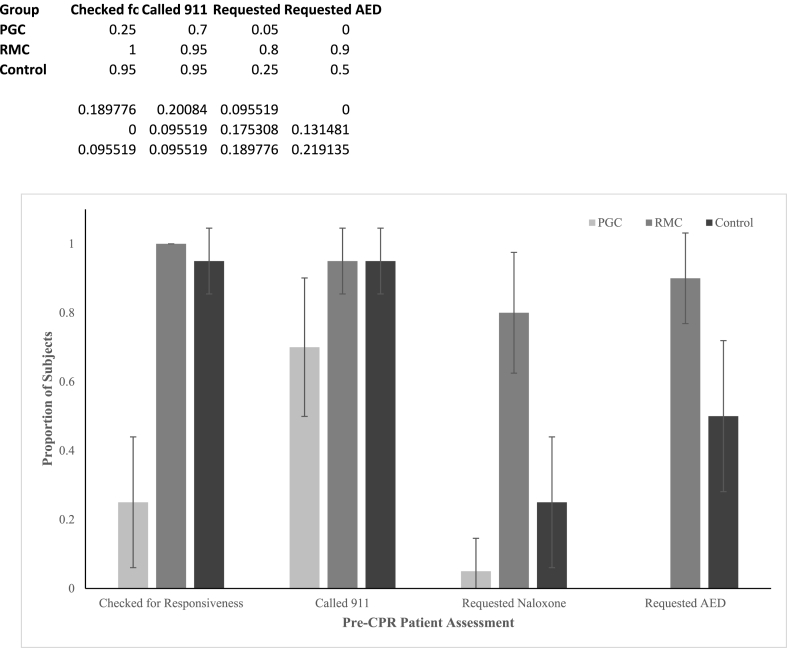
Rates at which control, ‘PG-CPR’ app using (PGC), and ‘Rescue Me CPR!’ app using (RMC) subjects performed pre-compression cardiopulmonary resuscitation (CPR) protocol steps. Error bars are 95 % confidence intervals.

The CPR-certified control group, PGC group, and RMC group each contained 20 subjects. Fig. 4 shows the rates each group performed key pre-compression steps in the CPR protocol. Subjects using the PGC app checked patient consciousness less frequently and with a greater delay, called 911 with a greater delay, requested an AED less frequently, and equivalently (i.e. rarely) asked for narcan, as compared to CPR certified subjects. They checked for consciousness 25 % (p < 0.01) of the time, called 911 70 % (p = 0.09) of the time, requested an AED 0 % (p < 0.01) of the time, and requested naloxone 5 % (p = 0.18) of the time. They assessed the patient and called 911 with respective delays of 30.3 s (p < 0.01) and 42.3 s (p < 0.01) as compared to CPR-certified subjects. RMC app users assessed patient conciousness and called 911 at equivalent rates, and requested both an AED and Narcan more frequently than CPR certified subjects. They checked for patient consciousness 100 % (p = 1.0), called 911 95 % (p = 1.0) of the time, requested an AED 90 % (p = 0.01) of the time, and requested naloxone 80 % (p < 0.01) of the time. They did these tasks with no statistically significant delay as compared to the control group. CPR-certified subjects checked for patient consciousness 95 % of the time, called 911 95 % of the time, requested an AED 50 % of the time, and requested naloxone 25 % of the time. An AED was not provided during this study, so its proper usage by each group was not studied. All subjects which requested naloxone, except 1 PG-CPR user, administered it properly once it was provided.

**Fig. 5:**
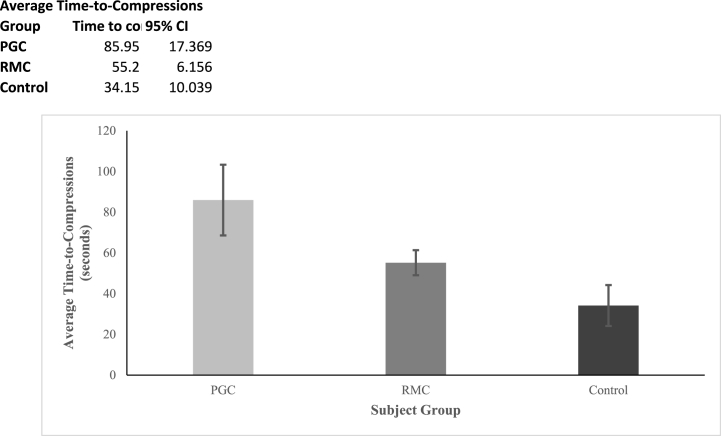
Average time to first compression in CPR performed by ‘PG-CPR’ (PGC), ‘Rescue Me CPR!’ (RMC), and control group subjects. Error bars are 95 % confidence intervals.

The average time from the first contact of the simulated victim to the start of compressions for each study group is shown in Fig. 5. CPR-certified subjects were faster to start chest compressions compared to the group using PGC and RMC apps: 34 ± 10 s compared to 86 ± 17 (p < 0.01) sec and 55 ± 6 (p = 0.04) sec respectively (average ± 95 % confidence interval).

Cycle times for rounds of compressions/ventilations (30:2) are an important metric to deliver oxygen to tissues, with reduced times indicating less non-perfusion time. We measured cycle times for the first three cycles per subject, but variance in how subjects concluded the fourth cycle (when breathing and circulation returned) made calculating cycle time for the fourth cycle impossible. Subjects using the PGC app performed the first, second, and third cycle of CPR in 44 ± 11, 28 ± 7, and 27 ± 6 s. Subjects using the RMC app accomplished the CPR cycles in 36 ± 3, 30 ± 3, and 30 ± 3 s. CPR-certified subjects did the same in 35 ± 8, 36 ± 10, and 32 ± 8 s respectively.

The higher the CCF, the more blood is circulated to vital organs, theoretically increasing the chance of survival. This is an important surrogate metric of vascular perfusion, with the AHA describing a CCF of 0.6 as sufficient and 0.8 or above as ideal [29]. Average CCF values for CPR certified group and group using the RMC app are shown in Table 1. Our data collection method did not account for the group using the PGC app to frequently pause and restart compressions each cycle, so we are unable to report an accurate CCF for this group.

**Table 1:** Chest compression fraction (CCF), modified CCF, and fractional achievement of a >0.8 CCF for subjects using the ‘Rescue Me CPR!’ (RMC) app and CPR certified subjects. CCF is the proportion of time spent performing compressions compared to the total duration of CPR during an out of hospital cardiac arrest. Modified CCF is the average ratio of time after a subject performed the first compression in which compressions are actively being performed, assessing CPR equally regardless of the delay to first chest compression. Ratio of CCF >0.8 is the fraction of subjects in each group which achieved a CCF greater than 0.8, the AHA's target for high-quality CPR.

Group	Total Compression Time (s)	CCF	Modified CCF	Fraction CCF >0.8
**RMC**	80	0.48	0.70	0.50
**Control**	71.71	0.52	0.66	0.25
***p-value***		*0.32*	*0.35*	*0.05*

The rate of CPR which meets the criteria of ‘high-quality’ (as defined above) is a key metric of CPR effectiveness. CPR-certified subjects had an aggregate rate of high-quality CPR compressions of 25 %. In other words, 25 % of cycles of CPR performed by this group meets ‘high-quality’ criteria. These same rates for PGC users and RMC app users were 2.5 % (p < 0.01) and 50 % (p < 0.01) respectively. Though, it should be noted that only 10 % of CPR certified subjects and 15 % (p = 0.49) of RMC app users performed high-quality CPR in all four compression cycles. Cycle-specific rates of high-quality CPR are shown in Table 2. While not a formal component of ‘high-quality’ CPR criteria, 0 % of control, 20 % of PGC and 10 % RMC app users elected to skip the rescue breaths step.

**Table 2:** Rate at which subjects in each study group performed high quality CPR in each cycle of CPR and in all four cycles together. P values for the ‘PG-CPR’ (PGC) and ‘Rescue Me CPR!’ (RMC) groups are reported relative to the control group's average performance in that cycle.

Group	Cycle 1	Cycle 2	Cycle 3	Cycle 4	All cycles
**PGC**	**0.0**	**0.0**	**0.0**	**0.10**	**0.0**
**RMC**	**0.45**	**0.55**	**0.45**	**0.55**	**0.15**
**Control**	**0.20**	**0.25**	**0.25**	**0.30**	**0.10**
**PGC p-value**	***0.11***	***0.05***	***0.05***	***0.24***	***0.49***
***RMC p-value***	***0.18***	***0.11***	***0.32***	***0.20***	***1.00***

**Fig. 6:**
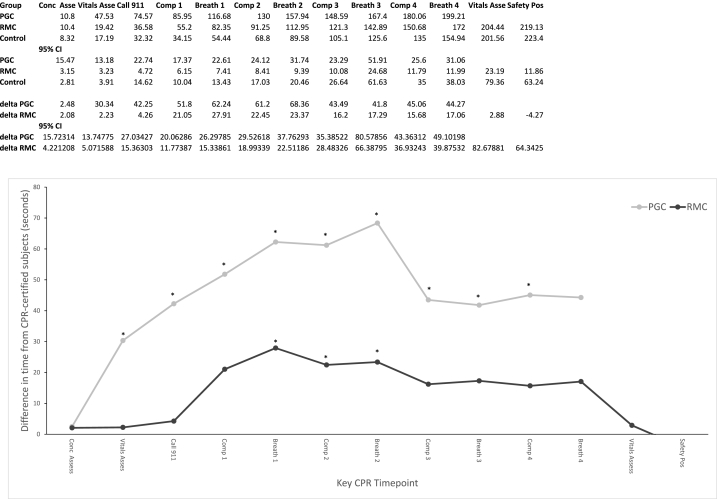
Relative timing of RMC app users and PGC app users achieving each key step of the CPR algorithm compared to CPR-certified subjects reaching the same steps. Steps included conciousness (Conc) assessment, vitals assessment, calling 911, and 3 cycles of compressions (Comp) and rescue breaths (Breath), followed by another vitals assessment and placement of the subject in a safety position (Pos). Timepoints indicated by * are statistically significantly increased from that of CPR-certified subjects (p = 0.05).

For CPR-GAs specifically, delay in CPR performance has been previously reported as a significant limitation in effectiveness [[20], [21], [22], [23]]. To report a measure of CPR delay, we assessed the relative timing to key steps of CPR between CPR certified subjects and the study's experimental groups. This data can be seen in Fig. 6. Overall, it demonstrates that both app using groups experience time delay in steps prior to, and during the first cycle of CPR. Thereafter, they either match or outpace CPR certified subjects at performing tasks. The RMC group experiences significantly less time delay compared to the PGC group.

## 4 Discussion

Over the last 60 years in the United States, there have been numerous concerted efforts to disseminate CPR training throughout the population. A properly designed smartphone could be a clever means of achieving that goal. To date, no study has validated the effectiveness of CPR guidance applications available within the USA. We previously developed a CPR guidance application and this study was performed to test its effectiveness at facilitating the performance of CPR by novices [24].

BLS delays brain damage but does not treat the underlying pathology in action. So, a key component of any CPR assistance technology is that it helps obtain resources like emergency medical responders, an AED, and naloxone, which each might resolve the situation. Our study demonstrates that while subjects using the most popular android CPR guidance app infrequently request such resources, nearly all the users of the RMC app requested EMS activation by dialing 911 at rates equivalent to CPR certified subjects. RMC app users also requested AEDs and naloxone at significantly higher rates than even CPR certified subjects. The use of each is associated with appreciable increases in OHCA survival [15]. From the perspective of acquiring crucial life-saving on-site resources for BLS, the RMC app is at least as good as formal CPR training. This is doubly important for novices, as they otherwise would have no way of knowing the value of or need to ask for an AED or naloxone. We suspect this high efficacy is most likely due to the app having a view explicitly dedicated to ‘coaching’ the user to acquire resources, where previously trained CPR providers might omit this step in the rush to perform compressions. As displayed in Fig. 6, these instructions, given prior to the ‘Call 911’ step only delay care by a few seconds, if any at all.

Reducing delay in the start of CPR, maximizing the ratio of time the patient is being perfused via compressions (i.e. Chest Compression Fraction), and increasing the overall quality of CPR have all been linked with increased OHCA survival rates [20,28,30]. The RMC and PGC app users in this study respectively initiated CPR compressions ∼20 s and ∼50 s slower than CPR certified subjects, though both reach compressions multiple minutes faster than the ∼3 min delay reported in dispatcher-assisted CPR, which somewhere between 20 % and 50 % of precincts don't provide data [[31], [32], [33]]. While delay in compression start is not ideal, it was least in RMC app users. As shown in Fig. 6, the delay in the PGC app occurs at every step prior to the start of compressions, whereas the delay in the RMC app occurs only immediately prior to the first cycle of compressions, when subjects are being given compression instructions. Further optimization in content delivery may be possible to shave off seconds before compressions start, but some delay at this step is unavoidable, as users are being taught a complex skill they previously may not know.

Once compressions are started, our data shows that users of the RMC app and CPR certified subjects perform CPR with similar CCFs, at ∼0.5 if calculated from first patient contact and ∼0.7 if calculated from first compression. Chest compression fraction, CCF is a key metric quantifying CPR effectiveness, with a value of 1.0 representing the perfect but impossible situation of uninterrupted compressions applied immediately upon seeing the patient, with zero interruptions. AHA guidelines recommend a CCF of at least 0.6 and an idealized CCF of 0.8 or greater [28,30]. So, neither CPR certified subjects nor RMC app users achieved ‘idealized’ CPR on average. However, on average both reached the minimum recommendations. RMC app users achieved ‘idealized’ CCF twice as often as the CPR-certified group. This surrogate for perfusion time suggests that RMC app users reach equivalent if not superior levels of perfusion once compressions begin. An even more comprehensive metric of BLS quality is the frequency of high-quality CPR, as defined by the AHA, as detailed above [28]. While no group studied performed high-quality CPR consistently, RMC users did in 50 % of aggregate CPR cycles, and CPR certified subjects did so in 25 % of cycles. Taken collectively, the results of this study demonstrate that in all key metrics, except time-to-first-compression, CPR provided by individuals using the RMC app is statistically equivalent or superior to CPR performed by a CPR certified individual. Contrastingly, in almost every metric, users of the PG-CPR app performed CPR inferior to CPR certified individuals. We hypothesize this improvement in quality is due to the constant ‘coaching’ of the app with visual, text and audio cues, but its exact mechanism requires further study.

There are multiple limitations of this study. First, the sample size of 20 per subject group resulted in appropriate statistical power to discriminate between the different studied groups but is not objectively large. Additionally, while exact age or demographic metrics were not recorded on subjects, they were all enrolled in either undergraduate or graduate education at the University of California San Diego between 2022 and 2023, suggesting a relatively young and educated cohort. It is well established that younger populations possess increased technological literacy, raising some uncertainties regarding the generalizability of this study's proposed app-effectiveness to all ages and socioeconomic groups. Further study into either the RMC app or other CPR-GA should use larger and more diverse sample sizes. Additionally, as this study only simulated medical emergencies and used a patient manikin, albeit a highly realistic one, subjects may have behaved more calmly and in compliance with app guidance than in a real medical emergency. Lastly, as we are both the designers of the ‘Rescue Me CPR!’ app as well as the researchers conducting this study, there is an inherent risk of observer bias. We took multiple steps to avoid this risk, including randomizing assignment of the subjects, recording objective metrics wherever possible, and outsourcing all data analysis to a researcher not involved in the development of the application and blinded as to which study group was which. We would also like to state here that the RMC app is free to download on the android and app store, advertisement free, and that we in no way profit from its downloading. That is not something we intend to change.

## 5 Conclusion

This study used medical simulation to shows the ‘Rescue me CPR!’ app, developed by this research team, can facilitate CPR as effectively as and possibly exceeds that performed by CPR-certified individuals. This was significantly better than the most downloaded CPR app on the Google play store. A smartphone application is not a substitute for formal AHA-compliant CPR training, but its easy access does make it a potentially valuable component of optimizing bystander OHCA care. We believe that this study objectively demonstrates that the ‘Rescue Me CPR!’ app can facilitate high-quality CPR, which could improve care in many incidents of OHCA which would be otherwise unassisted. Further, it is our hope that the results of this report inspire other groups to develop and study the effects and capabilities of CPR-GAs as well as have the AHA consider implementation of ‘Rescue Me CPR!’ or their own implementation of the technology to further disseminate life-saving CPR protocol to the masses.

## Funding

This research did not receive any specific grant from funding agencies in the public, commercial, or not-for-profit sectors.

## Ethics statement

This study was reviewed and approved by the 10.13039/100005522University of California, San Diego Kuali Institutional Review board ethics committee with the approval number 804734. All participants provided informed consent to participate in the study.

## Data availability statement

Data will be made available upon request made to the principal investigator, Dr. Mark Greenberg (mgreenberg@health.ucsd.edu).

## CRediT authorship contribution statement

**Brennan P. Marsh-Armstrong:** Writing – review & editing, Software, Methodology, Investigation. **Eri Seng:** Writing – review & editing, Formal analysis. **Fan Ting-Wei:** Writing – review & editing, Methodology. **Saka Stella:** Writing – review & editing, Project administration, Investigation. **Mark Greenberg:** Writing – review & editing, Writing – original draft, Visualization, Methodology, Conceptualization.

## Declaration of competing interest

The authors declare that they have no known competing financial interests or personal relationships that could have appeared to influence the work reported in this paper.
